# Enhanced topical cutaneous delivery of indocyanine green after various pretreatment regimens: comparison of fractional CO_2_ laser, fractional Er:YAG laser, microneedling, and radiofrequency

**DOI:** 10.1007/s10103-020-02950-2

**Published:** 2020-01-27

**Authors:** Marilin J. Nieboer, Arne A. Meesters, Mitra Almasian, Giota Georgiou, Menno A. de Rie, Rudolf M. Verdaasdonk, Albert Wolkerstorfer

**Affiliations:** 1 Netherlands Institute for Pigment Disorders, Department of Dermatology, Academic Medical Center, University of Amsterdam, Meibergdreef 9, NL-1105 Amsterdam, AZ Netherlands; 2 Department of Biomedical Engineering and Physics, Academic Medical Center, University of Amsterdam, Meibergdreef 9, NL-1105 Amsterdam, AZ Netherlands; 3 TechMed Center, BioMedical Photonics and Imaging group, University of Twente, Drienerlolaan 5, 7522 Enschede, NB Netherlands; 4 Department of Dermatology, Academic Medical Center, University of Amsterdam, Meibergdreef 9, NL-1105 Amsterdam, AZ Netherlands; 5 Department of Dermatology, VU Medical Center, De Boelelaan 1117, VU University, NL-1081 Amsterdam, HV Netherlands

**Keywords:** Drug delivery, Fractional laser, CO_2_ laser, Er:YAG laser, Microneedling, Radiofrequency

## Abstract

Different devices have been used to enhance topical drug delivery. Aim of this study was to compare the efficacy of different skin pretreatment regimens in topical drug delivery. In six ex vivo human abdominal skin samples, test regions were pretreated with fractional CO_2_ and Er:YAG laser (both 70 and 300 μm ablation depth, density of 5%), microneedling (500 μm needle length), fractional radiofrequency (ablation depth of ± 80–90 μm), and no pretreatment. The fluorescent agent indocyanine green (ICG) was applied. After 3 h, fluorescence intensity was measured at several depths using fluorescence photography. Significantly higher surface fluorescence intensities were found for pretreatment with fractional Er:YAG and CO_2_ laser and for microneedling vs. no pretreatment (*p* < 0.05), but not for radiofrequency vs. no pretreatment (*p* = 0.173). Fluorescence intensity was highest for the Er:YAG laser with 300 μm ablation depth (mean 38.89 arbitrary units; AU), followed by microneedling (33.02 AU) and CO_2_ laser with 300 μm ablation depth (26.25 AU). Pretreatment with both lasers with 300 μm ablation depth gave higher fluorescence intensity than with 70 μm ablation depth (Er:YAG laser, 21.65; CO_2_ laser, 18.50 AU). Mean fluorescence intensity for radiofrequency was 15.27 AU. Results were comparable at 200 and 400 μm depth in the skin. Pretreatment of the skin with fractional CO_2_ laser, fractional Er:YAG laser, and microneedling is effective for topical ICG delivery, while fractional radiofrequency is not. Deeper laser ablation results in improved ICG delivery. These findings may be relevant for the delivery of other drugs with comparable molecular properties.

## Introduction

Pretreatment of the skin with an ablative fractional laser is an effective method to enhance topical drug uptake in the skin, and this fractional laser-assisted drug delivery has become a well-established treatment modality [1, 2]. This technique acts by the principle of fractional disruption of the epidermis, creating an array of microscopic holes in the skin through which topical drugs can be delivered, with minimal risk of scarring [3]. For ablative fractional laser-assisted topical drug delivery, mainly carbon dioxide (CO_2_) lasers and Erbium Yttrium Aluminum Garnet (Er:YAG) lasers are used. Fractional CO_2_ lasers are generally believed to produce a significant necrotic eschar and a thick coagulation zone surrounding each ablation channel, whereas Er:YAG lasers produce a thinner coagulation zone, which may facilitate drug permeation, but might also be associated with more exudate formation impairing drug uptake [4–6]. Beside ablative fractional laser, other fractional techniques, including microneedling and fractional radiofrequency, have been investigated for the purpose of cutaneous drug delivery [7, 8]. Microneedling mechanically disrupts the epidermis by insertion of a grid of microscopic needles, while radiofrequency techniques produce plasma sparks that induce fractional epidermal ablation. To date, studies directly comparing these different pretreatment techniques are rare [9].

Aim of this study was to compare the accumulation of the fluorescent agent indocyanine green (ICG) as a representative drug model, in ex vivo human skin after pretreatment of the skin with a fractional CO_2_ laser, fractional Er:YAG laser, microneedling, or fractional radiofrequency (RF) device.

## Materials and methods

### Study design

The study evaluated the accumulation of fluorescence of ICG in 5 × 5 mm test regions in ex vivo human abdominal skin samples pretreated with fractional CO_2_ laser, fractional Er:YAG laser, microneedling, or fractional RF. ICG was left in place for 3 h. After application time, distribution and fluorescence intensity of ICG in the skin were assessed using digital surface fluorescence photography. Main outcome parameter was fluorescence intensity of ICG in the intact skin.

### Skin samples

Samples were consisted of excised human abdominoplasty skin and were collected at the department of plastic surgery at the Slotervaart Hospital in Amsterdam. In total, six skin samples from six individual patients were used. The use of skin from the Slotervaart Hospital for these experiments was approved by AMC Medical Ethics Committee officials. As skin samples were anonymized, a formal review procedure of the protocol by the Medical Ethics Committee was not required. Skin samples were stored and prepared according to a standardized protocol published earlier [10].

### Lasers

**Fig. 1:**
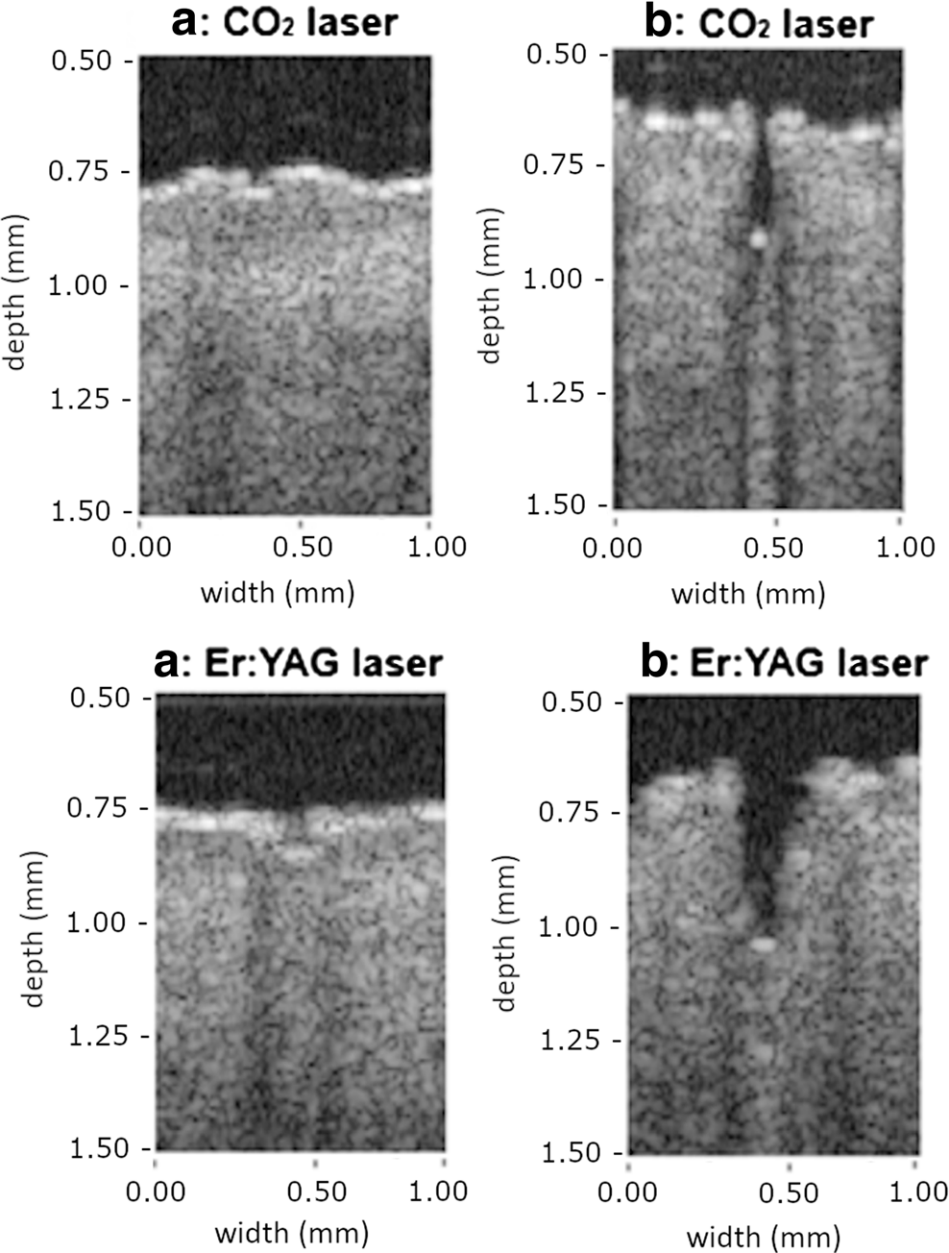
Optical coherence tomography images of a skin sample pretreated with the fractional CO_2_ laser at **a** 2.5 mJ/microbeam (single pulse) and **b** 20 mJ/microbeam (single pulse; microspot size 120 μm; pulse duration 80 μs) and with the fractional Er:YAG laser at **a** 9 mJ/microbeam (single pulse) and **b** 9 mJ/microbeam (nine stacked pulses; microspot size 225 μm, pulse duration 225 μs)

**Fig. 2:**
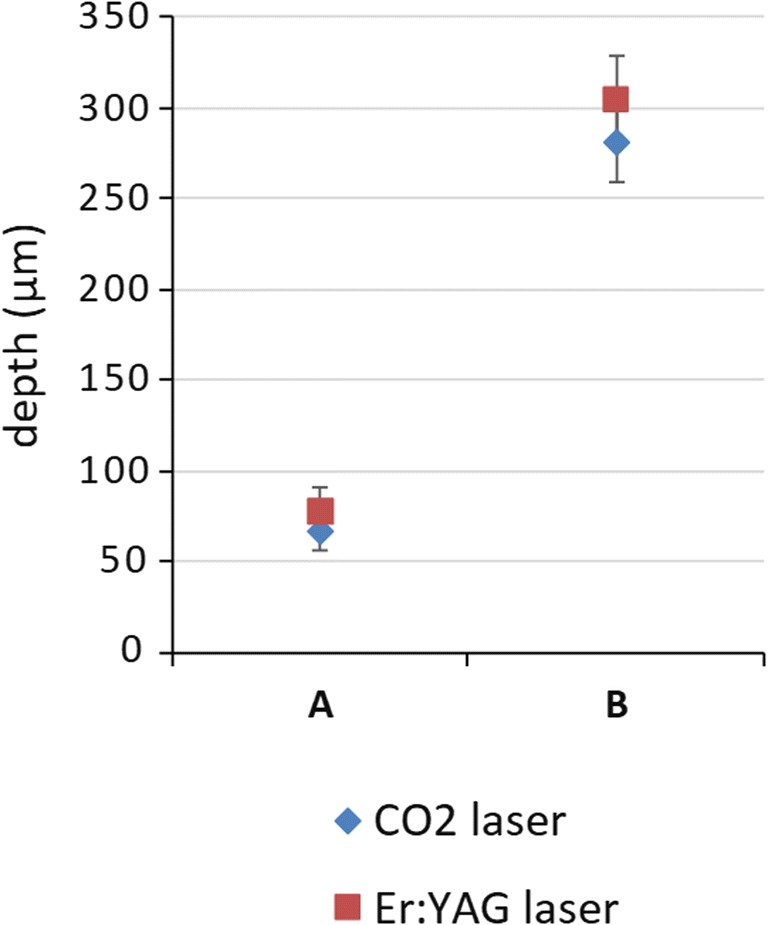
Ablation channel depth of the fractional CO_2_ and Er:YAG laser at two different settings, as assessed by three optical coherence tomography measurements (displayed as median and range). Settings were matched to achieve ablation channels of 70 and 300 μm depth. **a** CO_2_ laser, 2.5 mJ/microbeam (single pulse; microspot size 120 μm; pulse duration 80 μs); Er:YAG laser, 9 mJ/microbeam (single pulse; microspot size 225 μm, pulse duration 225 μs). **b** CO_2_ laser 20 mJ/microbeam (single pulse; microspot size 120 μm; pulse duration 80 μs), Er:YAG laser 9 mJ/microbeam (nine stacked pulses; microspot size 225 μm, pulse duration 225 μs)

Two fractional ablative lasers were used: a fractional 10,600 nm CO_2_ laser (Ultrapulse®, DeepFx handpiece; Lumenis Inc., Santa Clara, CA, USA) and a fractional 2940 nm Er:YAG laser (P.L.E.A.S.E.® Professional, Pantec Biosolutions, Rugell, Liechtenstein). Pretreatment with the CO_2_ and Er:YAG laser were compared at two different pulse energy settings corresponding to two different predetermined laser channel depths (Fig. 1), 70 μm and 300 μm. Optical coherence tomography (OCT) was used in order to optimize settings that created laser channels of equal depths for both lasers (Figs. 1 and 2). 2D and 3D images were acquired using a commercial swept source system (Santec Inner Vision 2000, Santec Corporation, Komaki, Japan) operating at 1300 nm central wavelength at 50 kHz, with an experimentally determined axial resolution of 13 μm in tissue and lateral resolution of 25 μm. The OCT measurements were performed in triplicate for each laser setting.

The CO_2_ laser was set at 2.5 mJ/microbeam and at 20 mJ/microbeam (600 Hz; pulse duration 80 μs, density 5%, 1 pulse, scanned area 5 × 5 mm, microspot size 120 μm) to create channels of 70 μm and 300 μm depth, respectively. The Er:YAG laser was set at 9 mJ/microbeam (0.9 W, 1 pulse duration:225 μs, density 5%, scanned area 5 × 5 mm, microspot size 225 μm). To create channels of 70 μm and 300 μm depth, one or nine stacked pulses (stacking frequency 100 Hz) were used, respectively.

### Microneedling

As a microneedling device, the Dermaroller 180 was used, containing 180 titanium needles with a length of 500 μm. The microneedling device was rolled 8 times across the skin in vertical, horizontal, and oblique directions, according to the manufacturer’s instructions.

### Radiofrequency

For RF, two passes with the Legato^II^ Pixel RF ™ at 60 W (Alma Lasers Ltd., handpiece: rolling tip, Nuremburg, Germany) were used, according to the manufacturer’s instructions. Ablation depth at these settings is approximately 80–90 μm and ablation width 220 μm [11].

### Indocyanine green

The fluorescent agent ICG (Cardiogreen, Sigma-Aldrich, St. Louis, MO, USA) was used as an indicator of drug absorption in the skin through the fractional laser ablation channels. ICG is a hydrophilic drug (Log P − 0.29) with a molecular weight of 775 Da. ICG was diluted with demineralized water to a concentration of 0.08 mg/ml according to the protocol published in our earlier study [10].

A total of 0.1 ml of ICG was occluded under a coverslip at each test region. During the application time, ICG was kept under dark circumstances by covering the skin samples with aluminum foil. At the end of the application time, excess ICG was wiped off from the skin.

### Fluorescence photography

Fluorescence of ICG in the skin was assessed using digital surface fluorescence photography performed in a dark environment to minimize exposure by ambient light. A 780-nm continuous wave diode laser was used as excitation source, and photographs were taken with a long pass cut-on 850-nm filter to block the excitation light and transmit an adequate amount of the fluorescence light, mounted on a full spectrum-modified digital camera (Sony α 5000, 24 mm objective, F/6,5, ISO 800, 10 s shutter speed) with a fixed focal distance. In order to achieve uniform light distribution of the diode laser on the test region, a diffuser was used. A reference point was incorporated in the corner of each photograph in order to correct for frame-to-frame variations. The reference point is consisted of a synthetic white diffusing bead on top of a fiber. The other end of the fiber was attached to an LED with an 810-nm peak emission generating a constant brightness at a fixed current/voltage.

Fluorescence images at skin surface, at 200 μm, and at 400 μm depth were collected. In order to enable imaging of deeper skin layers, the superficial skin layer was removed at the end of the application time by administering a pulse train with the CO_2_ laser (Acuspot 712, kamami tonsil tip, Lumenis Inc., Santa Clara, CA, USA) at the following settings: focused, tonsil, spot size 2, depth 1, 25 (200 μm ablation depth), and 60 W (400 μm ablation depth). OCT was used to verify depth of the ablated skin layers at various laser settings. The Acuspot 712 at the chosen settings provided a uniform removal of skin layers with minimal carbonization. Carbonized skin could be easily wiped off with a nonwoven gauze.

### Image analysis

**Fig. 3:**
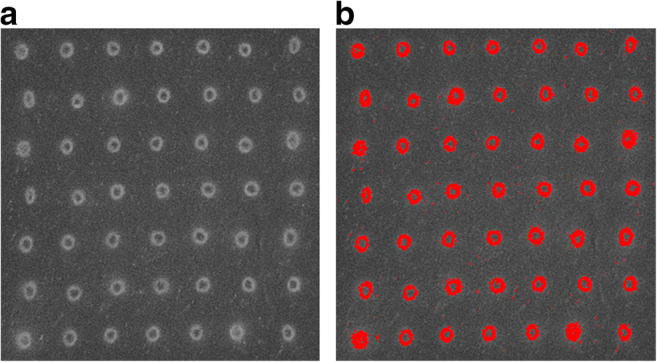
Surface fluorescence at a test region pretreated with the fractional CO_2_ laser (20 mJ/microbeam; single pulse; microspot size 120 μm; pulse duration 80 μs). **a** Standard surface fluorescence image. **b** Extreme values of fluorescence intensity are filtered out (red) after visual determination of a cut-off value to measure mean fluorescence intensity in the intact skin

Fluorescence images were analyzed using ImageJ (ImageJ 1.50i, National Institute of health, Bethesda, MD, USA). Fluorescence intensity was assessed at the skin surface, at 200 μm (upper reticular dermis), and at 400 μm (lower reticular dermis) depth by measuring gray value on a 0 to 255 arbitrary scale (0 representing black, 255 representing white). Mean fluorescence intensity was measured within a standardized circular area slightly smaller than the test region diameter (for the measurements in deeper skin layers, slightly smaller than the diameter of the full surface ablated area). Since the main outcome parameter was the fluorescence intensity in the intact skin (and not in the laser-induced coagulation zones surrounding the ablation channels, where mean fluorescence intensity is much higher), an additional measurement was performed for each test region pretreated with fractional CO_2_ and Er:YAG laser. In these additional measurements, the extreme values of fluorescence intensity found in the coagulation zones were filtered out by visual determination of a cut-off value of fluorescence intensity above which the measured values were excluded from calculation of the mean fluorescence intensity (Fig. 3). Images were corrected for autofluorescence of the skin by subtraction of the fluorescence intensity of the control image (no pretreatment, no ICG) at the skin surface, at 200 μm, and at 400 μm depth. Frame-to-frame variations were corrected for by the reference point.

### Statistical analysis

The statistical analysis of the outcomes was performed using Statistical Package for the Social Sciences 23 (SPSS, Chicago, IL). All data were entered in the SPSS database. Means, medians, and interquartile ranges (IQR) were calculated. Wilcoxon signed rank test was used to compare paired data. The significance level was set at *P* < 0.05.

## Results

### Visual assessment of fluorescence

In the non-pretreated regions, surface fluorescence was very weak (Fig. 3). In the CO_2_ and Er:YAG laser pretreated regions, fluorescence was more intense and was mainly concentrated around the ablation channels, especially in the regions pretreated with the more aggressive laser energy settings. We assume that these areas of intense fluorescence correspond with the laser-induced coagulation zones. As stated above, fluorescence intensity in these supposed coagulation zones was excluded from the measurements of mean fluorescence intensity per test region. In the regions pretreated with microneedling, overall fluorescence also appeared more intense compared to the regions that did not receive pretreatment. The pattern of the insertion points of the needles could still be recognized, but fluorescence was less pronounced compared to the coagulation zones surrounding the CO_2_ and Er:YAG laser channels. In the RF pretreated sites, some irregular patchy areas of increased fluorescence were observed, whereas overall fluorescence was weak.

**Fig. 4:**
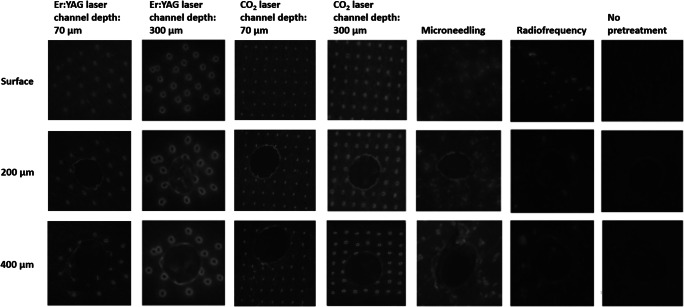
Fluorescence photographs of one of the skin samples at the skin surface, 200 μm depth and 400 μm depth. Imaging of the deeper skin layers was performed after removal of the superficial skin layers with a non-fractional CO_2_ laser. On the images, this ablated zone is represented by the circular area in the center of the test regions. Fluorescence is more intense in the test regions pretreated with fractional Er:YAG laser, fractional CO_2_ laser, or microneedling compared to unpretreated control and fractional radiofrequency. Fluorescence is most pronounced in the test regions pretreated with the fractional Er:YAG laser with an ablation channel depth of 300 μm

Removal of the superficial layer of the skin with the non-fractional CO_2_ laser enabled us to visualize the skin at various levels in the reticular dermis dependent on the laser settings (Fig. 4). At 200 μm depth, the pattern of the 300 μm deep CO_2_ and Er:YAG laser ablation channels ([CO_2_ 300]; [Er:YAG 300]) was visible, with fluorescence mainly concentrated in the supposed coagulation zone. No channel pattern was visible at 200 μm depth at the test regions pretreated with the CO_2_ laser at low energy settings (70 μm ablation depth; [CO_2_ 70]) with microneedling and with RF. The lowermost part of the coagulation zone of a few channels could be identified in some regions treated with the Er:YAG laser at low energy settings (70 μm ablation depth; [Er:YAG 70]). At 400 μm depth, the channel pattern could only be vaguely recognized in some of the [Er:YAG 300] test regions.

### Assessment of fluorescence intensity

#### Skin surface fluorescence

**Fig. 5:**
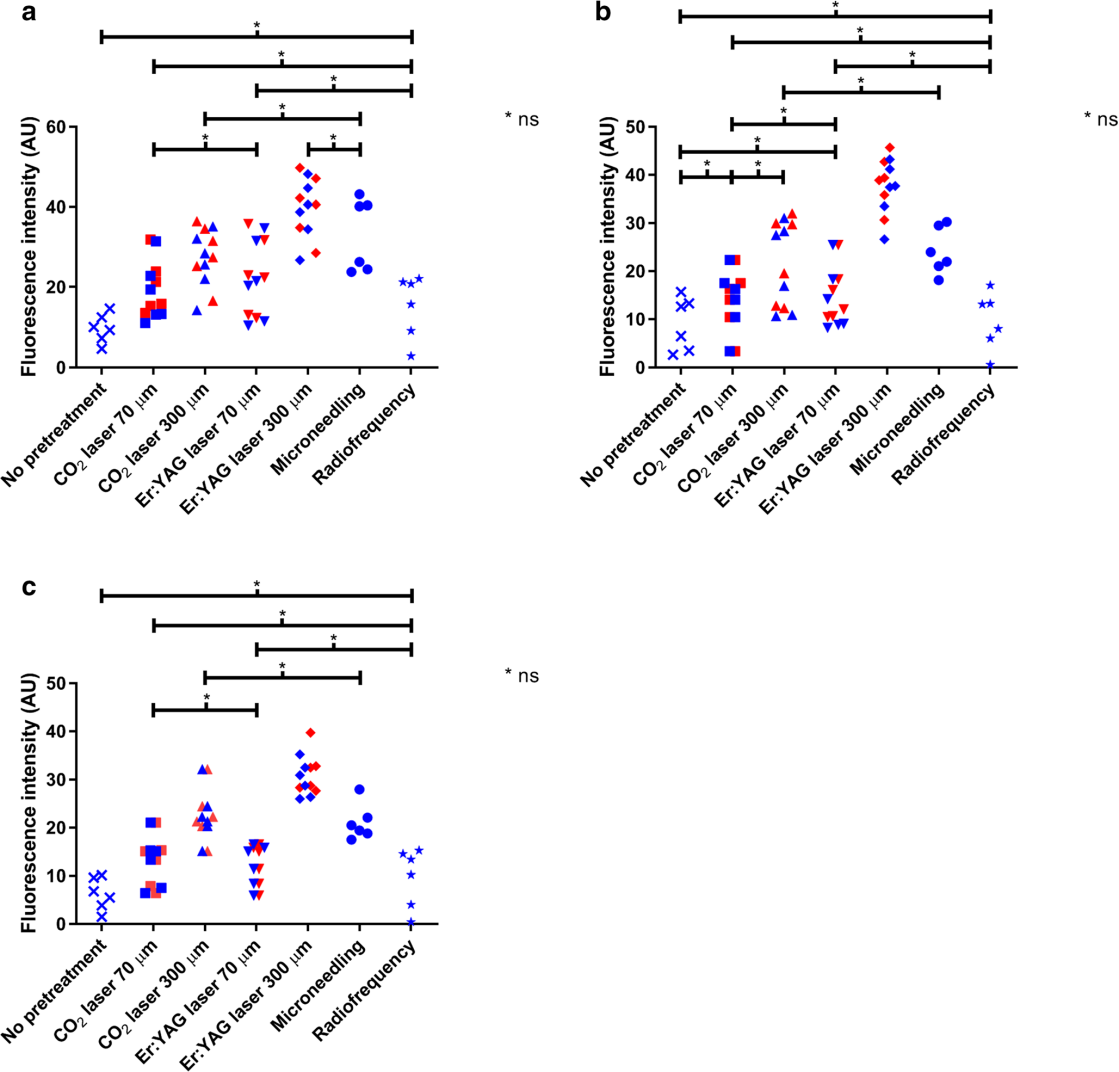
Fluorescence intensity of indocyanine green in arbitrary units (AU) after various pretreatment regimens at **a** the skin surface, **b** 200 μm depth, **c** 400 μm depth

Mean fluorescence intensities are displayed in Table 1. Fluorescence intensity was significantly higher for all test regions receiving any sort of pretreatment (Fig. 5a), compared to the control regions that did not receive pretreatment (10.40–48.19 arbitrary units [AU] vs. 4.65–14.64 AU; *p* < 0.05), except for RF (2.84–22.06 AU; *p* = 0.173). Mean fluorescence intensity was highest for the test regions pretreated with [Er:YAG 300], followed by microneedling, [CO_2_ 300], [Er:YAG 70], [CO_2_ 70], and RF, respectively. Fluorescence intensity was significantly higher for [Er:YAG 300], [CO_2_ 300], and microneedling compared to [Er:YAG 70] and [CO_2_ 70] and for [Er:YAG 300] compared to [CO_2_ 300] (*p* < 0.05).

**Table 1:** Mean fluorescence intensities (minimum-maximum) in the intact skin in arbitrary units (AU) after various pretreatment regimens and application times at various depths in the skin

	Channel depth (μm)	Surface (AU)	200 μm (AU)	400 μm (AU)
No pretreatment		9.70 (4.65–14.64)	9.02 (2.64–15.63)	6.22 (1.48–10.10)
CO_2_ laser	70	18.50 (11.02–31.44)	13.97 (3.35–22.32)	13.09 (6.41–21.02)
	300	26.25 (14.21–35.13)	20.86 (10.62–31.01)	22.59 (15.11–32.15)
Er:YAG laser	70	21.65 (10.40–34.69)	13.98 (8.19–25.42)	12.16 (5.89–16.47)
	300	38.89 (26.75–48.19)	36.57 (26.62–43.18)	29.94 (25.97–39.68)
Microneedling	± 500	33.02 (23.75–43.15)	24.11 (18.10–30.21)	21.03 (17.49–27.95)
Radiofrequency	–	15.27 (2.84–22.06)	9.64 (0.56–17.00)	9.61 (0.38–15.22)

#### Deeper skin layers

Both at 200 and 400 μm depth, fluorescence intensity tended to be significantly higher for the test regions receiving fractional laser or microneedling pretreatment, compared to the regions that did not receive pretreatment (3.35–43.18 AU vs. 1.48–15.63 AU; *p* < 0.05; Fig. 5b and c). At the test regions pretreated with RF, no increase in fluorescence intensity (0.38–17.00 AU) was observed compared to the non-pretreated control regions (200 μm depth, *p* = 0.917; 400 μm depth, *p* = 0.345). Fluorescence intensity was highest for the test regions pretreated with [Er:YAG 300, followed by [CO_2_ 300] and microneedling, followed by [Er:YAG 70] and [CO_2_ 70], followed by RF. [Er:YAG 300] rendered higher fluorescence intensity compared to all other pretreatment methods. Fluorescence intensity was significantly higher for [Er:YAG 300], [CO_2_ 300], and microneedling compared to [Er:YAG 70] and [CO_2_ 70] (*p* < 0.05; except for [CO_2_ 300] vs. [CO_2_ 70] at 200 μm depth: *p* = 0.075).

## Discussion

Our findings show that pretreatment with fractional CO_2_ laser, fractional Er:YAG laser, or microneedling enhances accumulation of ICG in the skin. We saw no significant increase in ICG accumulation following pretreatment with a fractional radiofrequency device. Low fractional laser energy settings, associated with very superficial ablation channels, appeared to be sufficient to achieve increased ICG accumulation, although ICG delivery was more effective through deeper channels. With the equipment and laser parameters used in this study, the Er:YAG laser that stacked several pulses appeared to be most effective. Microneedling with 500 μm long needles also resulted in increased accumulation of ICG, comparable to pretreatment with the fractional CO_2_ laser creating 300 μm deep ablation channels.

Studies comparing different ablative fractional laser modalities for the purpose of fractional laser-assisted drug delivery are still scarce. In 2018, we published a clinical study on fractional laser-assisted topical anesthesia, showing that pretreatment with fractional CO_2_ laser and fractional Er:YAG laser was comparably effective. With the low energy settings used in that study (channel depth 70 μm), these results are in line with our current findings. Classically, CO_2_ lasers are believed to create a wider coagulation zone than Er:YAG lasers since the 10,600 nm wavelength is less effectively absorbed by water than the 2940 nm wavelength [4]. However, we would like to stress that this dogmatic approach is often not feasible in practice, as ablation channel characteristics and coagulation zone width appear to be primarily determined by the combination of laser parameters chosen, especially pulse energy, pulse duration, and number of stacked pulses. According to an earlier study by Taudorf et al., the Er:YAG laser that was also used in the present study creates a coagulation zone of 30–40 μm at settings comparable to the 9 × 9 mJ/microbeam pulse energy settings used in our study [12]. According to earlier preliminary reflectance confocal microscopy images made at our institute, the fractional CO_2_ laser at 20 mJ/microbeam pulse energy induced coagulation zones that were of a similar width of 30–40 μm. In recent years, the importance of the fractional laser induced coagulation zone has been recognized. Where the coagulation zone was initially seen as a troublesome barrier for drug penetration, recent studies show that the presence of a thin coagulation zone of 20 μm actually facilitates drug delivery to the viable tissue [6, 13]. A thin coagulation zone prevents oozing and bleeding and may function as a reservoir through which drugs may be gradually released into the tissue.

In our present study, the fractional Er:YAG laser appeared to be more effective for the delivery of ICG than the fractional CO_2_ laser, when using relatively high energy settings (ablation channel depth: 300 μm). Possible explanations for this difference remain speculative. Although we were able to match the density and ablation channel depth between the CO_2_ laser and the Er:YAG laser, there were substantial differences in ablation channel width due to several factors including differences in microspot size (120 vs. 225 μm, respectively), more effective ablation at the Er:YAG laser wavelength (2940 nm), and possibly collapse of the ablation channels that are created in an explosive fashion by the ultrapulsed CO_2_ laser. As the Er:YAG laser creates larger ablation channels that may contain a larger volume of ICG, hydrostatic pressure of ICG within each channel is higher, whereas capillary action is smaller, which may facilitate penetration of ICG from the ablation channels into the skin. In earlier in vivo studies, microscopic regions of horizontal dissection have been observed after irradiation with pulsed mid-infrared lasers caused by laser-induced vapor bubble expansion. These regions can only be visualized after several days, when they are filled with blood [14]. Theoretically, this mechanism may be more important when using a laser that stacks several pulses, generating repeated micro bubbles. Subsequently, these dissected regions may make the skin more permeable for ICG, leading to higher tissue concentrations. This may also explain the differences in fluorescence intensity after pretreatment with the fractional Er:YAG laser and microneedling. In a study by Nguyen and Banga [7] assessing delivery of methotrexate after pretreatment with the same fractional Er:YAG laser as used in our study and a microneedling device, a similar difference between both modalities was found. The authors of this study acknowledge that both Er:YAG laser pretreatment and microneedling enhance skin delivery of methotrexate, although laser pretreatment delivered a significantly higher amount of drug into the skin. Interestingly, an earlier in vivo study by Bay et al. [9], comparing pretreatment with fractional CO_2_ laser, non-ablative fractional Er:glass laser, microneedling, and microdermabrasion, showed that pretreatment with fractional CO_2_ laser was more effective for delivery of protoporphyrin IX than microneedling. It should however be noted that in vivo microneedling may cause more bleeding or oozing of tissue fluid because of the absence of a coagulation zone. This may impair drug delivery and explain the suboptimal results compared to fractional CO_2_ laser pretreatment. Moreover, in microneedling, the skin is only disrupted without the formation of true ablation channels, as in fractional laser ablation. Therefore, microneedle-induced skin defects may be more likely to collapse in vivo compared to our tissue model where the skin was kept under constant tension.

Pretreatment with fractional laser resulted in much higher ICG fluorescence intensities than pretreatment with fractional RF. Fluorescence intensity at the test regions pretreated with RF was not significantly higher compared to unpretreated control. Studies showing increased drug delivery following fractional RF pretreatment of the skin go back as far as 2003 [8, 15, 16]. However, as far as we know, no previous studies exist comparing fractional RF with fractional laser for the purpose of drug delivery. Although ablation depth and width corresponded with the 1 × 9 mJ/microbeam settings of the Er:YAG laser, we observed slightly higher fluorescence intensity after Er:YAG laser irradiation at these settings than after fractional RF treatment [11, 12]. This might be due to the thick coagulation zone (relative to ablation channel depth) for the RF treatment (±50 μm) compared to that for the Er:YAG laser treatment (<± 25 μm) [11, 12]. We also noted that the RF device generated a very inhomogeneous pattern of ablation zones, where a large proportion of these zones could hardly be recognized, both before and after ICG application. The fractional lasers seemed to produce a more reproducible pattern of ablation channels. In fractional radiofrequency, thermal and biological effects may highly depend on local variations in electrical impedance due to the presence of blood vessels, hair follicles, and other adnexal structures.

When comparing the influence of ablation channel depth on ICG accumulation in the skin, we observed higher ICG fluorescence with 300 μm ablation channel depth than with 70 μm ablation channel depth. This is in accordance with other studies that suggested an ablation channel depth dependent uptake of hydrophilic substances, such as ICG. Deeper ablation channels are rapidly filled with interstitial fluid, providing a more favorable environment for hydrophilic than for hydrophobic drugs [17–19]. Also, deeper channels can simply contain a larger volume of the solution and have a larger contact surface with the surrounding skin, which probably enhances drug uptake.

Main limitations of this study are the ex vivo setting, the small number of skin samples, and the lack of histological controls. The skin samples all had been frozen for several days before being used for the experiments, because it was not possible to perform the experiments in freshly excised skin for logistic reasons. Surface fluorescence photography offers limited options for quantitative measurements and creates pitfalls for the interpretation of fluorescence intensity because of differences in optical properties of the structures visualized. For example, increased scattering of the light might be observed in the disintegrated tissue of the coagulation zone causing a risk for overestimation of ICG uptake. However, we were able to compare a large number of parameters in a reproducible way, while histology is sensitive to sectioning errors and unwanted alterations in tissue dimensions during fixation procedures [20, 21]. Histologic evaluation of fractional laser channels, especially when combined with fluorescence microscopy, is extremely time-consuming, thus limiting the number of parameters that can be compared or the number of skin samples that can be evaluated.

As stated in our previous study [10], only depths of up to 400 μm are investigated. Therefore, no conclusions can be drawn regarding the deposition of ICG in deeper skin layers. In addition, clinical applications for ICG are very limited. We used ICG merely as an indicator, and it might not be possible to extrapolate our findings to other drugs. Local pharmacokinetics might however be comparable to that of other hydrophilic drugs that may be used for fractional laser-assisted drug delivery, with a comparable molecular weight, including methotrexate (Log P, − 1.85; molecular weight, ±500 Da) [22, 23]. Since virtually all topical drugs that are commercially available are optimized for application on the intact skin, providing gradual release of the active substance through the stratum corneum, the development of new drug formulations exclusively for the purpose of fractional laser-assisted (or microneedling assisted) drug delivery is mandatory for the successful clinical application of this technique. In addition, physical penetration enhancement techniques, such as massage or ultrasound techniques, may be used to further enhance topical drug delivery in clinical practice [10].

In conclusion, pretreatment with fractional CO_2_ and Er:YAG laser enhances ICG accumulation in the skin. In our study setting, pretreatment with a fractional Er:YAG laser that stacks several pulses was more effective than pretreatment with a single pulse fractional CO_2_ laser, even when ablation channel depth and laser density are the same. Microneedling appeared to be a simple and effective alternative, although this might be different in vivo due to bleeding and oozing. Fractional RF did not seem effective. In line with earlier research, deeper ablation channels may be more effective for the delivery of a hydrophilic drug such as ICG. Further research is needed to correlate our findings to the histological effects of all pretreatment regimens and to reproduce our findings in vivo.
